# DFT studies of the role of anion variation in physical properties of Cs_2_NaTlBr_6*−x*_Cl_*x*_ (*x* = 0, 1, 2, 3, 4, 5 and 6) mixed halide double perovskites for optoelectronics

**DOI:** 10.1098/rsos.241680

**Published:** 2025-04-30

**Authors:** Mohammed Mehedi Hasan, Nazmul Hasan, Alamgir Kabir

**Affiliations:** ^1^ Department of Theoretical Physics, University of Dhaka, Dhaka 1000, Bangladesh; ^2^ Department of Electrical and Computer Engineering, North South University, Dhaka, Bangladesh; ^3^ Electrical and Computer Engineering, University of Rochester, Rochester, NY 14620, USA; ^4^ Department of Physics, University of Dhaka, Dhaka 1000, Bangladesh

**Keywords:** density functional theory, inorganic double perovskite, mixed halide perovskite, optoelectronics, anion doping

## Abstract

Halide double perovskites have various benefits over lead-based perovskites due to their suitable optical absorption efficiency, higher stability, tunable bandgap, large carrier mobility, easy availability and low cost. The structural, electrical, optical and mechanical characteristics of the lead-free halide double perovskites Cs_2_NaTlBr_6−*x*_Cl_*x*_ (*x* = 0, 1, 2, 3, 4, 5 and 6) are investigated by utilizing the first-principles density functional theory (DFT). The structural properties were computed at equilibrium, revealing that the crystals undergo structural phase transitions as the doping concentration varies. However, crystal stability was confirmed through the evaluation of the tolerance factor. The Heyd–Scuseria–Ernzerhof (HSE06) functional is used to correct the bandgap underestimation by generalized gradient approximation with Perdew–Burke–Ernzerhof (GGA-PBE). The band edge profile and electron density of states confirm the direct-bandgap semiconducting nature of the compounds. The bandgap increases approximately linearly with Cl incorporation, sharply tuned from 0.80 to 1.75 eV (GGA-PBE) and 1.78 to 2.98 eV (HSE06), making them highly suitable for photovoltaic and optoelectronic applications. The effective mass of the electron ranges from 0.283 to 0.449 m_0_, and the carrier mobility is from 83.913 to 305.485 cm^2 ^V^–1 ^s^–1^, respectively, suggesting excellent charge transport characteristics, essential for high-performance solar cells and photodetectors. The considered NaTl-based double perovskites have strong optical absorption in the visible and UV spectrum, with enhanced conductivity as Cl content increases. Binding energy analysis confirms strong lattice stability, with values decreasing from −3.193 eV (Br-rich) to −3.559 eV (Cl-rich), indicating improved thermodynamic robustness as Cl concentration increases. Mechanical analysis shows the ductile behaviour of the considered perovskites, supported by Poisson’s ratio and Pugh’s modulus. The rising bulk modulus and Debye temperature with Cl incorporation enhance rigidity and thermal stability. These insights advance the anion-engineered design of stable, high-efficiency, lead-free NaTl-based perovskites for next-generation optoelectronic applications.

## 1. Introduction

Perovskites are excellent candidates for sustainable and renewable energy applications, such as photovoltaics, owing to their growing power conversion efficiency (PCE) from 3.8% as reported in 2009 [1] to 26.1% in 2023 [2,3] when they are used as active materials in solar cells. The remarkable intrinsic characteristics of perovskite solar cells, including their large amount of visible-light absorption [4,5], small binding energy of exciton [6,7], long carrier diffusion length [8,9], long carrier lifetime and high carrier mobility [10,11], are the reason for their large value of PCE. These materials are essential to the scientific community to meet the increased global energy demands over traditional energy sources and climate change challenges [12]. However, the low stability of the materials is one of the main challenges to commercializing perovskite for photovoltaic applications [13]. Therefore, double perovskites (DPs), a similar geometrically shaped compound derived from the ideal perovskite structure ABX_3_, are the potential candidate to address these issues because of their high stability [14,15]. A DP has a general formula of A_2_BB′X_6_, where A represents a larger element (Cs or Rb), B and B′ are monovalent and trivalent cations, respectively (B = Na^+^, Ag^+^, Cu^+^; B′ = Bi^3+^, Sb^3+^, Tl^3+^, In^3+^), and X represents a halide ion (X = F^−^, Cl^−^, Br^−^) [14,16], and these materials have low carrier effective masses and highly tunable bandgaps in the range of visible light spectrum.

Halide DPs show both indirect and direct bandgaps, and several studies on different combinations of DPs, such as Cs_2_AgBiBr_6_, Cs_2_AgBiCl_6_, Cs_2_NaSbCl_6_ and Cs_2_CuSbCl_6_, have reported indirect bandgaps [17–20]. Atomic doping in B′ site by Sb in Bi leads to phase transition in double halide perovskites, which in turn tunes electronic bandgap values from indirect to direct nature for desired optoelectronic properties, where the concentration of dopant also influences the bandgap values from 2.46 to 3.09 eV [18]. In another study [19], authors addressed that as the valence state of bismuth is different from that of lead, that shrinks the possibility of replacing lead by bismuth in halide perovskites; hence, double halide perovskites based on Na were proposed, where density functional theory (DFT)-based calculations revealed that NaBi-/NaSb-based compounds demonstrated indirect bandgap values in all cases. However, they observed flat band dispersion in valence band maxima on account of varying halogen atoms for their studied perovskites, where I- and Br-based compounds exhibited optically prominent within the visible photon energy range. On the other hand, direct bandgap semiconductors are used in photovoltaic applications because they have slight energy loss, are more readily inspired and are better capable of completing the photoelectric conversion [21]. Furthermore, experimental studies [22,23] and theoretical study [24] on the double halide perovskite Cs_2_AgInCl_6_ have shown excellent stability, fast photo-response and low dark current, indicating their potential for customization for specific optoelectronic applications. Utilizing the advantage of self-trapped exciton emission, tunable dual emission covering visible light spectrum, enhancing energy transfer dynamics, was observed in Cs_2_NaInCl_6_ double halide perovskite by Sb and Mn doping resolving the indirect bandgap issue in such perovskites [25,26], the DPs Cs_2_AgTlX_6_ (X = Cl and Br) have shown direct bandgaps of 2.0 and 0.95 eV, respectively [27]. Although the Cs_2_InBiCl_6_ and Cs_2_InSbCl_6_ structures are two of the few candidates that have the potential to provide promising electronic properties, including direct bandgaps and smaller carrier effective masses, they demonstrate less feasible ability to produce stable semiconducting substances [28]. Recently, a new theoretical study has reported the stability of Cs_2_NaTlBr_6_ DP with a direct bandgap of 1.82 eV, which can be a suitable candidate for photovoltaic solar cells [29]. Mixed halide DPs Cs_2_AgBiBr_6−*x*_Cl_*x*_ show bandgap tunability with an indirect nature after incorporating Cl atoms, which made this material more stable and viable in photoelectronic applications [30,31].

To date, there has been a lack of comprehensive and comparative studies investigating the effect of halogen substitution on the properties of NaTl-containing double halide perovskites. However, studies on AgBi-based mixed halide DP found them more stable and suitable for photoelectronic applications [30,31]. Despite the promising optoelectronic properties of lead-free halide DPs, there remains a lack of systematic studies on the effect of anion substitution on their structural, electronic and optical properties in such NaTl-based DP phases. To bridge the existing research gap, this study systematically investigates the structural, electronic, optical and mechanical properties of mixed-halide DPs Cs_2_NaTlBr_6−*x*_Cl_*x*_ (*x* = 0−6) using first-principles DFT. The generalized gradient approximation with Perdew–Burke–Ernzerhof (GGA-PBE) functional was employed to evaluate fundamental material properties, while the hybrid Heyd–Scuseria–Ernzerhof (HSE06) functional was applied to obtain more accurate bandgap estimations, addressing the known GGA-PBE underestimation issue. Our analysis reveals a systematic increase in bandgap with higher Cl incorporation, correlated with the reduction in halogen atomic size, as confirmed by the curve fitting of bandgap variations. Structural assessments confirm the thermodynamic stability of these compounds, whereas mechanical analysis indicates varying degrees of ductility and rigidity, suggesting composition-dependent mechanical behaviour. This DFT-based study demonstrates that halogen engineering effectively tunes bandgap, charge transport and light–matter interactions, optimizing stability, optical absorption and mechanical properties for next-generation optoelectronic and photovoltaic applications. Additionally, a comparative analysis with previous theoretical and experimental studies validates the reliability of our results, reinforcing the potential of NaTl-based double halide perovskites as promising lead-free alternatives for optoelectronic devices.

## 2. Computational methodology

The Vienna *ab initio* simulation package was employed to perform the computations in this research [32,33]. All the parameters are evaluated using the GGA [34] with PBE functional [35] in DFT [36]. Projector augmented wave [37] is considered to compute the interactions between electrons and ions. The HSE06 [38] hybrid functional is more accurate in describing the band structure of strongly correlated systems [39]. The HSE06 functional (25% of Hatree–Fock exchange) is used in this study for pure compounds to get the correct band structure. A convergence threshold of force was chosen as 0.001 eV Å^−1^, and the cut-off energy is 500 eV. Up until the self-consistent total energy was changed to 10^−8^ eV/atom, the whole unit cell’s ionic coordinates, shape and volume were utterly relaxed. The Brillouin zone integral was summed throughout the entire Brillouin zone using a K-mesh of Monkhorst–Pack 3 × 3 × 3 for all the Cl- and Br-based DPs. Since all the investigated crystal structures were fully optimized, the larger k-points and higher energy cut-off have little or no impact on the computed outcomes.

### 2.1. Physical behaviour of the mixed halide Cs_2_NaTlBr_6−*x*_Cl_*x*_ perovskites

#### 2.1.1. Structural properties

**Figure 1.**
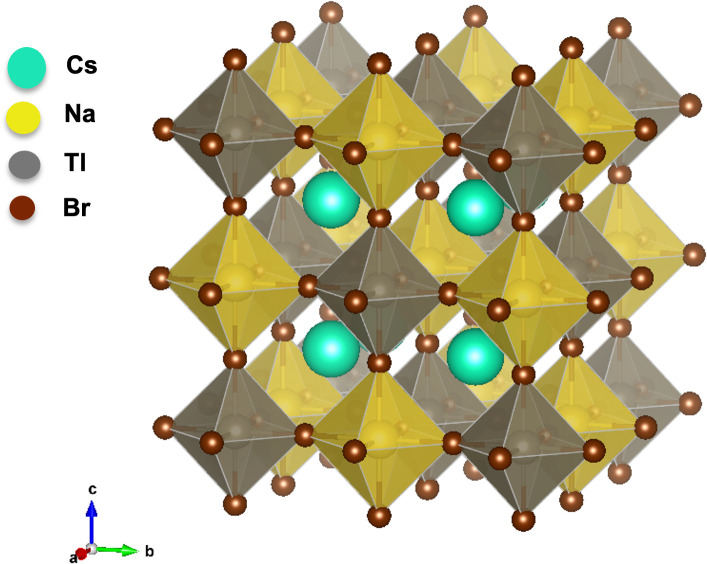
Geometrically optimized cubic crystal with Fm−3m space group of Cs_2_NaTlBr_6_ halide double perovskite.

The face-centred cubic (rock salt) crystal structure of Cs_2_NaTlBr_6_ is presented in figure 1, which belongs to the space group Fm−3m (225). We have substituted Br with Cl atoms in this compound at a different proportion in distinct positions and relaxed them, turning these materials into several crystal structures. The symmetry of the crystalline structure is reduced, and the crystal structure of Cs_2_NaTlBr_6−*x*_Cl_*x*_ (*x* = 1, 3) transforms to Amm2, and *x* = 2, 4, 6 turns to the Pm space group.

**Figure 2.**
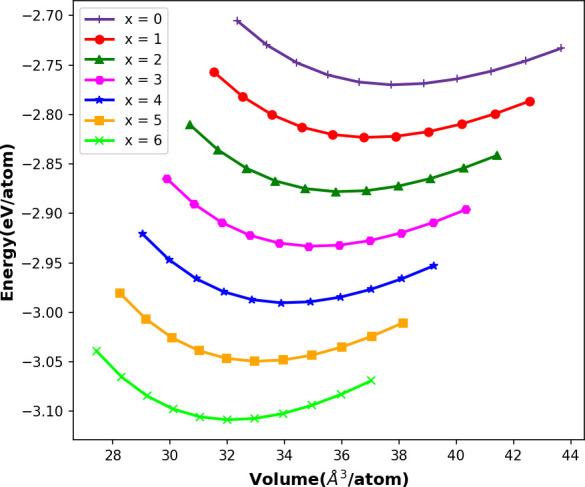
Computed energy variations by volume of the crystals for halide double perovskite Cs_2_NaTlBr_6−*x*_Cl_*x*_.

The equilibrium lattice parameter *a*_0_, total volume *V*_0_, total energy *E*_0_, the bulk modulus *B*_0_ and its pressure derivative *B*_0_′ using GGA-PBE functional are tabulated in table 1. The lattice parameter, total cell volume and pressure derivative of bulk modulus decrease with increasing concentration of Cl in the Cs_2_NaTlBr_6−*x*_Cl_*x*_ mixed-halide DPs. This can be attributed to the diminution of the atomic size of Cl atoms. On the other hand, the total energy and bulk modulus of the DP decrease with the increase of the atomic size. The equilibrium lattice parameter *a*_0_ (in Å) is compared with available experimental [40] and theoretical [41] data for the Cs_2_NaTlCl_6_ compound and found to be in good agreement. To the best of our knowledge, no experimental data are available so far for Cs_2_NaTlBr_6_ and other compounds. The total energy of the unit cell for different cell volumes is calculated and fitted to the energy per atom versus volume per atom curves in figure 2 using the third-order Birch–Murnaghan equation [42].

**Table 1.** Presented crystal system, space group of the materials and calculated lattice parameters (Å), total cell volume *V*_0_ (Å^3^), total energy *E*_0_ (eV), pressure derivative of bulk modulus *B*_0_′, tolerance factor *t*, formation energy Δ*E*_f_ (eV), binding energy *E*_b_ (eV), energy bandgap *E*_g_ (eV) and bandgap nature using GGA-PBE approach of Cs_2_NaTlBr_6−*x*_Cl_*x*_.

properties	*x* = **0**	*x* = **1**	*x* = **2**	*x* = **3**	*x* = **4**	*x* = **5**	*x* = **6**
crystal system	cubic	orthorhombic	monoclinic	orthorhombic	monoclinic	monoclinic	cubic
space group	Fm−3m	Amm2	Pm	Amm2	Pm	Pm	Fm−3m
lattice parameters (Å)	*a* = *b* = *c* = 11.47, 11.13 [29]	*a* = 11.339 *b* = 11.392 *c* = 11.390	*a* = 11.246 *c* = 11.251 *b* = 11.314	*a* = 11.171 *b* = 11.177 *c* = 11.166	*a* = 11.030 *b* = 11.090 *c* = 11.076	*a* = 10.884 *c* = 10.889 *b* = 11.119	*a* = *b* = *c* = 10.85, 10.62 [40], 10.58 [40]
*V*_0_ (Å^3^)	1516.71	1479.95	1441.94	1404.04	1364.98	1325.39	1286.76
*E*_0_ (eV)	−110.80	−112.93	−115.12	−117.32	−119.62	−121.98	−124.34
*B*_0_'	5.27	5.24	5.21	5.20	5.18	5.18	5.19
*t*	0.847	0.848	0.850	0.851	0.852	0.853	0.854
Δ*E*_f_ (eV)	−1.477	−1.476	−1.475	−1.475	−1.476	−1.477	−1.484
*E*_b_ (eV)	−3.193	−3.251	−3.311	−3.370	−3.432	−3.496	−3.559


(2.1) $$P\left(V\right)=\frac{3}{2}B_{0}\left[{\left(V_{0}/V\right)}^{\frac{7}{3}}-{\left(V_{0}/V\right)}^{\frac{5}{3}}\right]\;\left\{1+\frac{3}{4}\left(B_{0}^{\prime }\;-4\right)\left[{\left(V_{0}/V\right)}^{\frac{2}{3}}\;-1\right]\right\}.$$

The tolerance factor (*t*) usually estimates the stability of perovskites, $t=(R_{A}+R_{X})/\surd 2(R_{B}+R_{X})$, using the Goldschmidt rule, where *R*_*A*_ and *R*_*X*_ are the radii of the ions positioned at the A and B sites (Na and Tl in this case), respectively, and *R*_B_ is the average ionic radius of the elements at the B and B′ sites. A structure will be stable if the tolerance factor is within the range of 0.81 < *t* < 1.1 [43]. The tolerance factors for the Cs_2_NaTlBr_6−*x*_Cl_*x*_ compounds are found to be 0.847 to 0.854, respectively, as presented in table 1. All values of the tolerance factor *t* are within the range satisfying the stability criterion of the Goldschmidt tolerance factor, which indicates that all the studied compounds are stable.

The formation energy ($\Delta E_{f}$) of a compound is a measure of the thermodynamic stability of a material and is estimated by the following equation [44]:


(2.2) $$\Delta E_{f}=\;\left[E_{total}-\sum_{a}nE_{a}\right]/N,$$

where *n* is the number of atoms in the unit cell. For example, the formation energy for the Cs_2_NaTlBr_6−*x*_Cl_*x*_ compounds is given by the following formula:


(2.3) $$\Delta E_{f}(Cs_{2}NaTlBr_{6-x}Cl_{x})=[E_{Cs_{2}NaTlBr_{6-x}Cl_{x}}-8E_{Cs}-4E_{Na}-4E_{Tl}-xE_{Cl}-(24-x)E_{Br}]/40.$$

A negative value of the formation energy indicates that the formation of the compound is exothermic and, therefore, thermodynamically stable, as the energy released during the formation process is greater than the energy required to form the compound. Our calculated values of formation energies of all the Cs_2_NaTlBr_6−*x*_Cl_*x*_ compounds are presented in table 1, and it is found that the negative values of formation energy of all the studied compounds indicate that the compounds are thermodynamically stable and exothermic. The formation energy can also be related to the Gibbs free energy of formation, which is a measure of the spontaneity of reactions. In this case, the negative values of the formation energy indicate that these compounds can be formed spontaneously under standard conditions. This is in accordance with the thermodynamic principle of decreasing Gibbs free energy for spontaneous reactions [45]. The stability of these compounds can also be understood from the perspective of the crystal structure. The stability of a crystal structure depends on the strength of the chemical bonds between the atoms, which is a direct result of the electron–electron interactions in the system [46,47]. As the formation energy of these compounds is negative, it can be inferred that the electron–electron interactions in these compounds are favourable and lead to a stable crystal structure. To analyse the strength of the electrons’ interaction within the crystal and understand the stability, we determined the binding energies (*E*_b_) through the following relation [48]:


(2.4) $$E_{b(Cs_{2}NaTlBr_{6-x}Cl_{x})}=\frac{E_{Cs_{2}NaTlBr_{6-x}Cl_{x}}-8\mu_{Cs}-4\mu_{Na}-4\mu_{Tl}-x\mu_{Cl}-(24-x)\mu_{Cl}}{40},$$

where $E_{Cs_{2}NaTlBr_{6-x}Cl_{x}}$ is the total energy of primitive cells, and *μ* is the chemical potential of a single atom. From table 1, values for binding energies are found as −3.193 eV for Br-contained perovskite, where −3.559 eV is found for Cl-based phases. The calculated binding energies of Cs_2_NaTlBr_6−*x*_Cl_*x*_ mixed-halide DPs are negative and decrease with the number of Cl atoms increasing. This indicates that these DPs can be synthesized, and their stabilities become strong Br- to Cl-based compounds. Moreover, the binding energy influences the position of valence and conduction bands of double halide perovskites. A smaller bandgap indicates a higher absorption of light and greater photoconductivity, whereas a higher conduction band minimum (CBM) and lower valence band maximum (VBM) suggest a better potential for charge separation and improved device performance. Besides, a larger bandgap limits the light absorption range but can improve the material’s efficiency by reducing recombination losses. Binding energy also influences the mobility of charge carriers, such as electrons and holes, within the material. More substantial binding energy tends to lower mobility, making it harder for the carriers to move through the material. This can limit the efficiency of optoelectronic devices, such as solar cells, as charge carriers may recombine instead of being collected at the electrodes. The binding energy can also affect the stability of metal halide perovskites, as weaker bonds are more susceptible to degradation and environmental factors. This can be a significant challenge in the development of metal halide perovskite-based optoelectronics, as the material’s performance may degrade over time.

#### 2.1.2. Electronic properties

**Figure 3.**
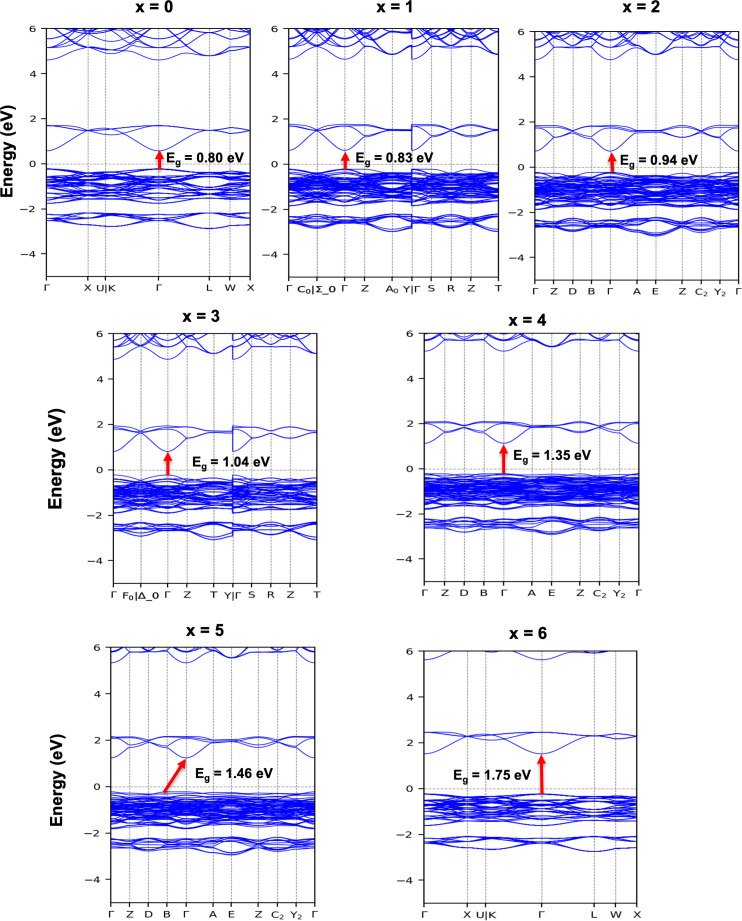
Calculated energy band structures for Cs_2_NaTlBr_6−*x*_Cl_*x*_ halide double perovskites: GGA-PBE executed profiles.

Metals, semimetals, semiconductors and insulators can be distinguished based on their electronic properties, and the computed electronic band structure can be used to recommend the materials under consideration for specific industrial applications. The electronic band structures for Cs_2_NaTlBr_6−*x*_Cl_*x*_, calculated using GGA-PBE functional, are shown in figure 3, and the bandgap values are reported in table 2. We have direct bandgap values ranging from 0.80 eV for Cs_2_NaTlBr_6_ to 1.75 eV for Cs_2_NaTlCl_6_, executing GGA-PBE functional at the centre of the Brillouin zone (Γ point). However, the Cs_2_NaTlBrCl_5_ compound shows only an indirect bandgap of 1.46 eV from B to Γ direction. It is clearly observed that the values of the bandgap or semiconducting nature increase with the decreasing radius of the halide ions from Br to Cl. This tendency might be caused by the decrease in the electronegativity difference between B′-site elements like Tl (1.62) and halide ions like Cl and Br (3.16 and 2.96, respectively), which strengthens the covalent bond between Tl and Cl/Br element. This, in turn, may push the Tl–Cl or Tl–Br bonding orbitals to the higher energy states, increase the energy of the VBM and reduce the bandgap of the materials. The direct bandgap semiconductors are expected to be suitable for photovoltaics and optoelectronic device applications [39,44,51,52].

**Table 2.** Estimated energy bandgap *E*_g_ (eV), bandgap nature, effective mass (*m*_e_*) and carrier mobility (*μ*_bulk_) of electrons of Cs_2_NaTlBr_6−*x*_Cl_*x*_ double perovskites.b

properties		***x* = 0**	*x =* **1**	*x* = **2**	*x* = **3**	*x* = **4**	*x* = **5**	*x* = **6**
*E*_g_ (eV) this work	GGA-PBE	0.80, 1.02 [49] 0.57 [29]	0.83	0.94	1.04	1.35	1.46	1.75
	HSE06	1.78, 1.827 [29] (mBJ + SOC) 1.85 [50] (TB-mBJ)						2.98, 3.10 [50] (TB-mBJ)
bandgap nature		direct	direct	direct	direct	direct	indirect	direct
*m*_e_*		0.283	0.297	0.386	0.345	0.449	0.439	0.437
*μ*_bulk_ (cm^2 ^V^–1 ^s^–1^)		294.062	254.496	305.485	166.553	83.913	85.874	86.631

**Figure 4.**
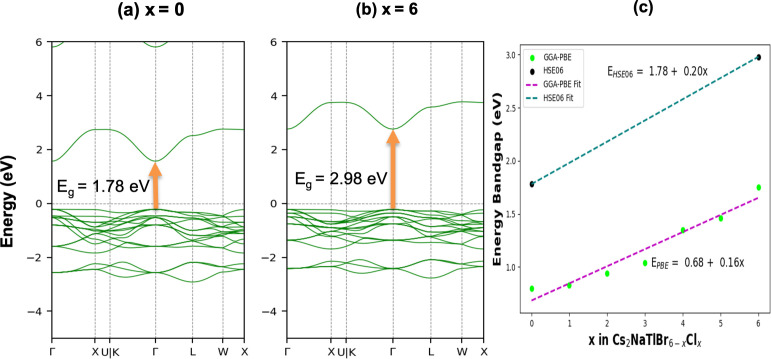
The calculated band structures of (a) *x* = 0, (b) *x* = 6 in Cs_2_NaTlBr_6−*x*_Cl_*x*_ using HSE06 functional, and (c) the trend of bandgap values and their fitted line with the ratio of Br and Cl in the Cs_2_NaTlBr_6−*x*_Cl_*x*_ double perovskites.


Since we were aware that GGA-PBE functional underestimates the bandgap values, we decided to calculate the bandgap using the hybrid functional (HSE06), as this functional computes the bandgap value more accurately, though it needs high computational assistance. It is important to note that the well-known self-interaction effect causes very noticeable differences in bandgap between the values as calculated by using GGA-PBE and HSE06 functional. The values of the bandgap for Cs_2_NaTlBr_6_ and Cs_2_NaTlCl_6_ are estimated to be 1.78 eV and 2.98 eV, respectively, within the HSE06 functional and are shown in figure 4. It is clear from the bandgap value that these two pure compounds behave like semiconductors. The bandgap values are higher in the HSE06 calculation than in the GGA-PBE calculations as the conduction bands shift towards higher energy values, which is evident from the depiction of figure 4c. The energy bandgap values and *x* of Cs_2_NaTlBr_6−*x*_Cl_*x*_ were fitted through the following equations:

$$E_{PBE}=0.68+0.16x$$

$$E_{HSE}=1.78+0.20x.$$

We get a clear idea of the bandgap values and their trends with the change of proportion Br/Cl in the crystal system. The obtained value of bandgaps has a linearly increasing trend with a slightly different slope value in the GGA-PBE and HSE06 methods.

The energy dispersion curve is fitted around the bottom of the conduction bands and the top of the valence bands to determine the effective mass (*m*_e_*) of electrons, which can be determined by the relation (2.5),


(2.5) $$m_{e}^{*}=\hbar^{2}{\left[\frac{\partial^{2}\varepsilon \left(k\right)}{\partial k^{2}}\right]}^{-1},$$

where *k* is the wave vector, and ε(*k*) is the band-edge eigenvalues. We calculated the effective mass of electrons *m*_e_* in the k space and presented it in table 2. High carrier mobility is essential for the high performance of optoelectronic devices. Br-based compounds possess smaller electron effective masses than the other materials, which means that this compound realized comparatively high carrier mobility for studied materials. In general, the carrier mobility is directly related to the electronic properties of materials, such as the band structure and the nature of the charge carriers. According to Bardeen and Shockley’s deformation potential theory, we can get carrier mobility $\mu_{bulk}$ in bulk materials from the following expression [53,54]:


(2.6) $$\mu_{bulk}=\frac{{\left(8\pi \right)}^{1/2}\hbar^{4}eC}{3\;{\left(m_{e}^{*}\right)}^{5/2}\;{\left(k_{B}T\right)}^{3/2}E_{d}^{2}},$$

where e is the charge of an electron, ħ is the reduced Planck constant, C is the elastic constant, *k*_B_ is the Boltzmann constant and *T* is the room temperature (*T* = 300 K). *m*_e_^*^ is the effective mass of an electron and *E*_d_ is the deformation potential of the CBM for electrons along the transport direction. The deformation potential is defined as *E*_d_ = Δ*E*_d_/ (Δ*V*/*V*_0_); here, Δ*V* is the deformation of a crystal under an appropriate unit compression or expansion, *V*_0_ is the lattice volume at equilibrium for a three-dimensional system and Δ*E*_d_ is the energy change of the CBM under an appropriate unit compression or expansion. The largest and smallest values of carrier mobilities are 305.485 and 83.913 cm^2 ^V^–1 ^s^–1^ for Cs_2_NaTlBr_4_Cl_2_ and Cs_2_NaTlBr_2_Cl_4_, respectively. The $\mu_{bulk}$ values of Br-dominant compounds are around three times or greater than the Cl dominant DPs.

This can be understood by considering the nature of the halogen elements (Br and Cl) in each compound. The halogen element acts as a dopant in these materials, introducing impurities that act as additional charge carriers. The larger the halogen element, the more impurities are introduced and the greater the number of charge carriers available to move through the material. Considering Br-based compounds, the larger atomic size of bromine leads to the introduction of more impurities and, hence, more charge carriers, resulting in higher carrier mobility compared with Cl-based compounds. This can be attributed to the larger ionic radius of the Br atom, leading to a more loosely packed crystal lattice and, thus, less scattering of carriers. The band structure calculations of these compounds also showed that the Br-dominant compound had a higher density of states near the Fermi level, indicating a higher carrier concentration and, thus, a higher mobility. These results suggest that the Br-dominant compound may have potential for use in electronic devices such as transistors and solar cells, where high carrier mobility is desired. It should be noted that this conclusion is based on theoretical calculations using DFT and the GGA-PBE method, and further experimental studies are needed to confirm the predicted order of carrier mobility in these materials. Other factors, such as structural defects, crystal quality, temperature, etc., also affect carrier mobility, which is essential to consider.

**Figure 5.**
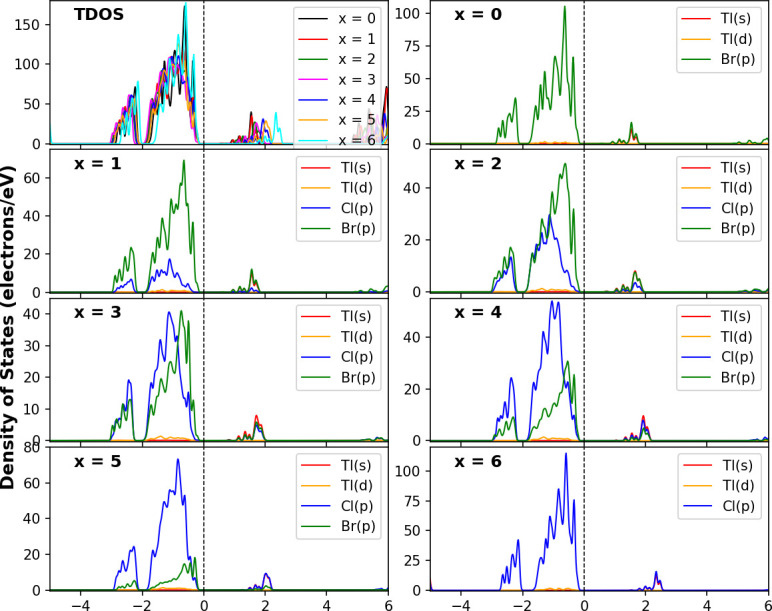
DFT calculated electronic properties with TDOS and PDOS of Cs_2_NaTlBr_6−*x*_Cl_*x*_ (*x* = 0, 1, 2, 3, 4, 5 and 6) halide double perovskites.

The total density of states (TDOS) and partial density of states (PDOS) of materials provide a more detailed insight into the orbital contributions to the VBs and CBs in the electronic structure. Figure 5 shows the computed TDOS and PDOS for Cs_2_NaTlBr_6−*x*_Cl_*x*_ with energies ranging from −5 to 6 eV. It is clear from figure 5 that the d orbital of the Tl atom (5d) and the p orbital of the halogen atom (3p for Cl and 4p for Br) contribute significantly to the top of the VB, whereas the wide 5s orbital of the Tl atom and the p orbital of the halogen atoms are the major contributors to the bottom of the CB. A small contribution of Tl 3d states to the top of the VB is observed, but it decreases gradually from Br to Cl element-dominated materials. The presence of Tl 5s in Cs_2_NaTlBr_6−*x*_Cl_*x*_ suggests that substituting the Cl ion with Br does not significantly impact the valence states of the Tl atom. However, the conduction states are shifted considerably to higher energy levels.

The perovskites Cs_2_NaTlBr_6−*x*_Cl_*x*_ possess a semiconducting nature and direct bandgap values in most compounds, rendering them suitable for various optoelectronic applications. The ability to tune band structures and bandgaps by altering the number of atoms at the halogen sites in halide DP materials containing NaTl has led to their prominence as photoelectric conversion materials.

#### 2.1.3. Optical properties

**Figure 6.**
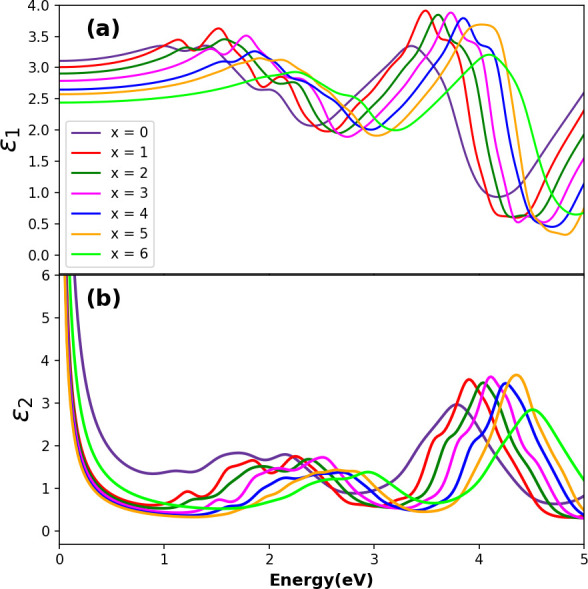
Optical properties for Cs_2_NaTlBr_6−*x*_Cl_*x*_ double perovskites: (a) real part (ε_1_) of complex dielectric function, (b) imaginary part (ε_2_) of complex dielectric function with respect to photon energy.

**Figure 7.**
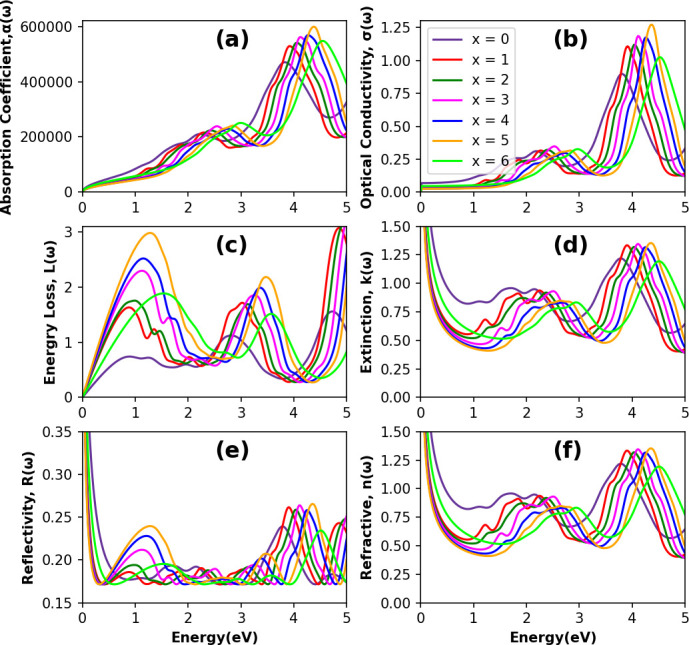
The evaluated (a) optical absorption coefficient α, (b) optical conductivity σ, (c) energy loss function L, (d) extinction coefficient k, (e) reflectivity R and (f) refractive index n for the Cs_2_NaTlBr_6−*x*_Cl_*x*_ mixed halide double perovskites.

Optical properties are the most important in determining a material’s suitability for optoelectronic and photovoltaic applications since they provide insight into how well a material interacts with light. To comprehend a material’s electronic configuration and assess its feasibility for optoelectronic applications, understanding the optical properties of a material is vital. This study examines the detailed optical characteristics of the considered double-halide perovskite materials, including complex dielectric function (ε) and the derived parameters named photo-absorption coefficient α(ω), reflectivity R(ω), refractive index n(ω), optical conductivity σ(ω), energy loss function L(ω) and extinction coefficient k(ω) corresponding to photon energy E (eV) of the electromagnetic spectrum. To analyse the optical properties of the Cs_2_NaTlBr_6−*x*_Cl_*x*_ (*x* = 0, 1, 2, 3, 4, 5 and 6) DP, we employed first-principles calculations in DFT. The profile of the absorption coefficient α(ω) is essential for determining the light-harvesting efficiency of the perovskite materials, which is governed by materials’ dielectric dispersion behaviour [55]. To understand the fundamentals of photogenerated carriers, polarization nature and the exciton binding energy with mixed halide compositions, the dielectric constants for the DPs are calculated and portrayed in figure 6. Complex frequency-dependent dielectric functions ε(ω) are determined by Cohen and Ehrenreich’s equation [56]. Electrical susceptibility and complex susceptibility can be determined using the dielectric function [57]. However, other necessary optical parameters, such as refractive index n(ω), optical conductivity σ(ω), energy loss function L(ω) and extinction coefficient k(ω) are also calculated using the result of the real and imaginary part of the dielectric function [58]. In figures 6 and 7, DFT-extracted optical parameters are represented for 0–5 eV of photon energy to comprehend reliably the light–matter interaction across the infrared to ultraviolet solar energy radiation in the electromagnetic spectrum of the considered perovskite phases in this study.

The complex dielectric function with the combination of real ε_1_(ω) and imaginary ε_2_(ω) parts is used to measure the dielectric nature of the materials. Since the optical properties of a crystal depend on the structure, hence, in turn, it depends on the band structures. The inter-band transitions significantly contribute to ε(ω) in semiconductor materials, whereas the intra-band contributes to the complex dielectric function for metal-type materials [58]. The real component ε_1_(ω) of the complex dielectric function is associated with anomalous dispersion and electronic polarization, whereas the imaginary component ε_2_(ω) is related to the optical absorption of compounds. Thus, the ε_2_(ω) spectrum reveals the process of electronic transitions between occupied and unoccupied states in the valence and conduction bands. The Kramers–Kronig transformation relations are used to calculate the real and imaginary components of the dielectric function [59]. The dipole matrix resulting from excitonic electron transitions determines the imaginary component ε_2_(ω) of the complex dielectric function; moreover, the Fermi distribution function of the valence band, the energy variation between the valence (*i*) and conduction (*f*) band states [δ(*E*_*f *_− *E*_*i* _− ħω)] at the k point due to photon absorption energy (ħω) linearly modulates the dielectric behaviour of the perovskite materials and hence a material’s other necessary optical properties [60].

From figure 6a, it is observed that ε_1_(ω) exhibits its maximum peak with the first harmonic after a gradual rise at 1.35 eV, where increasing the concentration of Cl in X site leads to the first peak’s right shift to photo energy. Notably, in the visible energy range, an increase in Cl content substituting Br at the X site of the Cs_2_NaTlBr_6−*x*_Cl_*x*_ perovskite crystal results in an initial increase in the magnitude of the real part of the dielectric function up to *x* = 3; as the Cl concentration exceeds 50% of the total site occupancy, the dielectric function magnitude decreases. This behaviour is attributed to octahedral distortions induced by variations in the atomic radii of the mixed halide elements within the crystal lattice. Additionally, in the UV region, it is observed that the magnitude of ε_1_(ω) increases with the Cl content until the X site is fully occupied with Cl atoms, which occurs because the B–X bond length increases due to the lower atomic radius of Cl, resulting in a decreased magnitude. The apparent variable dielectric behaviours with changing X-site compositions showcase higher transmittance in visible to UV energy regions where low transmittance is observed in visible and IR energy regions for the compositions above 50% occupancy by Cl. These fluctuations are influenced by factors including bandgap, electronic structure and inter-band transitions within the material’s optical properties. It is evident, based on both the imaginary and real portions of the dielectric function from figure 6, that increasing the Cl content with Br results in a higher amplitude depending on the mixing ratio in the X site. However, the static real dielectric constant, ε_1_(0) is linearly decreased from 3.13 to 2.45 with increasing the mixing ratio in the X site by Cl with Br, which indicates reduced polarizability in response to the field [54]. Moreover, no compound shows negative dielectric values in the real part of ε_1_(ω), ascertaining their semiconducting nature as previously confirmed by their band structure configurations [61], and hence no free electron oscillations-led surface plasmon polariton will appear. This positive real dielectric magnitude will be influencing the design and efficiency of optical devices by governing the refractive index in the device junctions. Concerning the imaginary dielectric function ε_2_(ω) as illustrated in figure 6b, the threshold values where ε_2_(ω) starts to increase are found to be a right shift from 0.85 eV (*x* = 0) to 2 eV (*x* = 6), which is also aligned with the electronic bandgap trend in figure 4c. Moreover, comparing with the absorption profile in figure 7a, all the absorption peak positions are accurately matched with the imaginary dielectric behaviour as shown in figure 6b. The matched behaviour of imaginary dielectric values with the electronic bandgap and absorption confirms that these NaTl-based double halide perovskites realize electronic excitation by photon energy occurring optical transition from the valence band to the conduction band at these threshold values [60].

The photo-absorption coefficient (α) quantifies light penetration and absorption within a medium, with figure 7a illustrating the photon energy-dependent absorption characteristics of Cs_2_NaTlBr_6−*x*_Cl_*x*_ DP compounds. The dielectric functions in figure 6b proportionally influence the absorption profile, and the first peak absorption profile aligns well with the fundamental energy bandgap shown in figures 3 and 4. Within the visible photon energy range (1.5−3.1 eV), the absorption profiles for all compositions steadily increase, with the first harmonic observed in the visible spectrum and major peaks appearing in the UV range around 4 eV. Increasing the Cl content in the mixed halogen site raises the absorption magnitude, peaking at 4.38 eV in the UV range for *x* = 5, while the absorption decreases in the IR energy range but starts to rise within the visible spectrum as Cl content changes. Substituting Br atoms with Cl at the X site induces a rightward shift in the absorption profile, illustrating the influence of halogen ions’ octahedral effect due to ionic radius variations. In effect, the tunable exciton binding energy can enhance carrier mobility in these perovskite-based devices by promoting efficient photoelectric conversion with visible light compared with other perovskites’ absorption profiles [62]. This demonstrated tunability enables the controlled design of NaTl-based DPs, offering significant promise for nano-optoelectronics.

Optical conduction in perovskite crystals entails electron transfer from valence to conduction bands via photon absorption, where photoconductive materials show enhanced electrical conductivity upon electromagnetic radiation absorption, quantified by σ(ω) and illustrated in figure 7b for the studied perovskite compounds. The absorption profile of perovskites in figure 7a directly correlates with their photoconductivity profiles in figure 7b due to photon absorption-induced electron excitation, augmenting charge carrier population and thereby enhancing the material’s conductivity. The optical conductivity profile (σ(ω)) for Cs_2_NaTlBr_6_ begins at 0.5 eV, whereas for Cs_2_NaTlCl_6_, it initiates at 1.5 eV, with a rightward shift corresponding to variations in Cl concentration. Across the photon energy spectrum of solar radiation (0.494–4.12 eV), all phases exhibit optical energy transport following photon absorption. Phase *x* = 5 (Br_6−*x*_Cl_*x*_) peaks highest at 4.3 eV, while *x* = 3 maximizes solar energy utilization in the visible range, suggesting superior photogeneration potential for device applications [63–66]. Figure 7c illustrates the energy loss function, evaluating electrons’ rapid energy loss effects on the optical properties of the studied perovskites across the solar photon energy range (0−5 eV). Increasing Cl content correlates with elevated electron energy loss in the perovskite. Energy loss functions in figure 7c reveal that these perovskites exhibit finite energy loss when photon energy surpasses the binding energy of a tightly bound molecule, as indicated by photo-absorption and conduction profiles. The extinction coefficient k(ω) serves as a critical optical parameter indicative of absorption losses and device efficiency, crucial for predicting the potential of materials in photovoltaic and other optoelectronic applications [67]. In figure 7d, the DFT-calculated extinction coefficient variation for photon energy is depicted. Within the visible spectrum, the extinction coefficient decreases as halogen ions with smaller ionic radii replace Br with Cl. Specifically, for *x* = 0, the coefficient reaches higher values of 0.9 in the visible energy range, reducing to 0.4 for *x* = 5. Notably, the Cs_2_NaTlBrCl_5_ perovskite exhibits a maximum peak of 1.35 at 4.25 eV across the entire spectrum. The systematic variation of the extinction coefficient guides the optimization of fabrication processes to minimize scattering losses and environmental sensitivity while controlling carrier recombination and optimizing charge transport, which enhances the generation of photo-excited charge carriers for robust optoelectronic devices. Reflectivity, a key optical parameter delineating light-surface interactions through the ratio of reflected to incident light energy provides insight into the surface properties of perovskite materials. The DFT-simulated reflectivity spectra for mixed halide DPs Cs_2_NaTlBr_6−*x*_Cl_*x*_, as depicted in figure 7e, show that the visible energy region for the considered double halide perovskites exhibit lower reflectivity, where with increasing Cl content, magnitude decreases. All Cs_2_NaTlBr_6−*x*_Cl_*x*_ phases exhibit consistently low reflectivity (less than 25%) across the solar photon energy spectrum, suggesting enhanced absorptivity and/or transmission capabilities [68]. The static reflectivity at zero frequency R(0) decreases with changes in halogen mixing. The observed low reflectivity in the visible energy range highlights the materials’ suitability for solar cell applications.

Understanding optical refraction is crucial for comprehending light propagation through materials; as the refractive index increases, light bends closer to the normal direction. Figure 7f depicts the calculated refractive index of double-halide Cs_2_NaTlBr_6−*x*_Cl_*x*_ perovskites as a function of solar photon energy. Similar to the behaviour of extinction coefficients in figure 7d, the refractive index of all the studied perovskites shows a consistent qualitative pattern. This uniformity in refractive index suggests predictable light–matter interactions, which is crucial for designing optoelectronic devices such as solar cells and light emitting diodes (LEDs). A consistent refractive index ensures efficient light absorption and minimal scattering losses, enhancing overall device performance. Moreover, the optical susceptibility (χ) of perovskite materials provides a quantitative measure of the response of the crystalline material’s dipole moment (P) to an external electromagnetic field, making it a fundamental quantity that influences the propagation of light through the material by affecting the refractive index and absorption coefficient. Table 3 displays the variations in susceptibility values due to the concentration variations of Br–Cl atoms in Cs_2_NaTlBr_6−*x*_Cl_*x*_ double halide perovskites. The optical susceptibility decreases as the Cl content increases. The smaller Cl ions create a more tightly packed crystal lattice, reducing the overall polarizability. Conversely, the larger Br ions result in a more distorted and polarizable lattice, contributing to higher optical susceptibility. Materials with a narrow bandgap and low exciton binding energy also tend to have higher optical susceptibility, resulting in a higher refractive index and stronger light absorption. Polarizability is related to the dielectric function and optical refraction through the Clausius–Mossotti and Lorentz–Lorenz [69,70], which affect the optical path and confinement in devices like waveguides and photonic crystals. In devices such as solar cells, LEDs and photodetectors, this means that the material’s response to light (effective light trapping, absorption, emission, etc.) is weaker with higher Cl content, whereas it might reduce unwanted reabsorption in LEDs, improving light extraction efficiency. Reduced optical susceptibility can lead to changes in the electric field distribution within the material, and this could influence charge transport efficiency and recombination dynamics, potentially influencing the behaviour of photoexcited carriers and affecting overall device efficiency.

**Table 3.** The calculated values of static dielectric constant Ɛ_1_(0), static refractive index n(0), static reflectivity R(0) and susceptibility (χ) for the studied double halide perovskites.

Cs_2_NaTlBr_6−*x*_Cl_*x*_ phases	Ɛ_1_(0)	n(0)	R(0)	χ
***x*** **= 0**	3.13	5.33	0.45	2.13
***x*** **= 1**	3.00	4.85	0.42	2.00
***x*** **= 2**	2.87	4.83	0.41	1.87
***x*** **= 3**	2.76	4.82	0.41	1.76
***x*** **= 4**	2.63	4.80	0.41	1.63
***x*** **= 5**	2.55	1.75	0.39	1.55
***x*** **= 6**	2.48	5.00	0.42	1.48

The estimated magnitudes of optical properties for Cs₂NaTlBr_6−*x*_Cl_*x*_ perovskites in our study align well with previous research on similar double halide phases. According to studies [66,71–73], absorption coefficients of 4−5 cm⁻¹, static dielectric constants of values ranging from 2 to 3 and refractive indices in the visible range of 5−9 are suitable for photovoltaic applications. Our results show that the Cl and Br phases of these perovskites are promising for energy-harvesting photovoltaic applications.

#### 2.1.4. Mechanical properties

**Figure 8.**
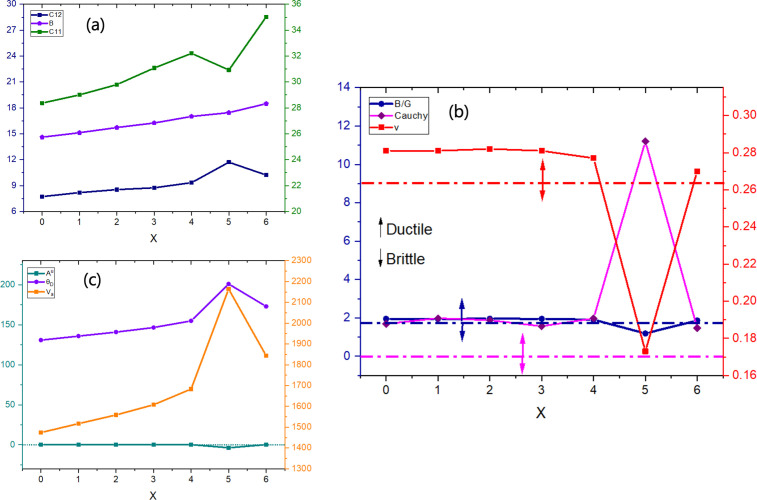
Mechanical behaviour of the Cs₂NaTlBr_6−*x*_Cl_*x*_ mixed-halide double perovskite varying with X-site atom compositions: (a) elastic constants and bulk modulus trend, (b) ductility and brittleness trend and (c) anisotropy, Debye temperature and average velocity variations.

For any crystalline solid, the elastic tensor provides a comprehensive insight into the material’s mechanical stability. In this study, finite strain theory was employed to calculate the elastic parameters of double halide Cs₂NaTlBr_6−*x*_Cl_*x*_ (*x* = 0–6) perovskites. Table 4 summarizes the stress–strain analysis results for elastic moduli (*C*_*ij*_) to evaluate the mechanical behaviours of these materials. The cubic symmetry of perovskite crystals should comply with the Born stability criteria: *C*₁₁> 0, *C*₄₄ > 0, *C*₁₁ − *C*₁₂ > 0 and *C*₁₁ + 2*C*₁₂ > 0 [74]. It is evident from table 4 that the studied perovskites Cs₂NaTlBr_6_ and Cs₂NaTlCl_6_ satisfied well with the criteria, indicating that these two phases are mechanically stable. In addition, these double halide perovskites under study satisfy the cubic crystal stability condition: *C*_12_ < B < *C*_11_ of the elastic tensor, as represented in figure 8a. The orthorhombic DPs Cs₂NaTlBr_3_Cl_3_ and Cs₂NaTlBr_5_Cl also form mechanically stable materials according to the Born stability criteria.

**Table 4.** Independent elastic constants or stiffness tensor *C*_*ij*_ (in GPa) and nature of mechanical stability of Cs_2_NaTlBr_6−*x*_Cl_*x*_ compounds.

properties	*x* = **0**	*x* = **1**	*x* = **2**	*x* = **3**	*x* = **4**	*x* = **5**	*x* = **6**
*C*_11_	28.369, 45.09 [29]	29.003	29.797	31.078	32.207	30.926	35.024, 41.27 [50]
*C*_12_	7.726, 10.98 [29]	8.194	8.533	8.748	9.340	11.706	10.218, 13.52 [50]
*C*_13_	—	8.194	8.877	8.983	9.611	9.536	—
*C*_22_	—	29.176	29.752	30.065	31.920	29.943	—
*C*_23_	—	8.012	8.963	9.846	9.455	11.468	—
*C*_33_	—	29.176	29.202	29.962	32.118	30.781	—
*C*_44_	6.035, 7.82 [29]	6.207	6.653	7.176	7.369	0.504	8.744, 5.27 [50]
*C*_55_	—	6.422	6.897	7.150	7.726	8.076	—
*C*_66_	—	6.422	6.677	6.940	7.650	2.013	—
*C*_15_	—	—	−0.069	—	−0.040	−0.709	—
*C*_25_	—	—	0.042	—	0.055	−0.057	—
*C*_35_	—	—	−0.050	—	0.027	0.740	—
*C*_46_	—	—	0.007	—	−0.031	−4.792	—
stability	stable	stable	unstable	stable	unstable	unstable	stable

In the case of three monoclinic crystals Cs_₂_NaTlBr_6−*x*_Cl_*x*_ (*x* = 2, 4, 5), one of the 12 elastic stability criteria does not match. The condition $C_{22}\left(C_{33}\times C_{55}\;-\;C_{35}^{2}\right)$ + $2\times C_{23}\times C_{25}\times C_{35}$ − $\left(C_{23}^{2}\right)\times C_{55}\;-\;\left(C_{25}^{2}\right)\times C_{33}\;>\;0$ does not meet for the Cs₂NaTlBr_4_Cl_2_ and Cs₂NaTlBr_2_Cl_4_ compounds, which declares the instability of the materials. Furthermore, as Cs₂NaTlBrCl_5_ DP does not agree with the condition $C_{44}\times C_{66}-\;C_{46}^{2}\;>\;0$, it can be referred to as an unstable compound for the monoclinic structure [74]. For more insights along with structural formation and mechanical stability, we computed the phonon band structures and explained them for all the considered compounds in this study (see electronic supplementary information).

Table 4 and figure 8a reveal that *C*_11_ is significantly higher than *C*_12_ and *C*_14_, indicating a high resistance to flexure along the [100] ([010] or [001]) direction and a lower resistance to shear deformation. This discrepancy can be attributed to octahedral distortion, which implies stronger coupling between the a and c crystal directions as compared with the a and b or b and c directions. To determine the brittleness and ductility of materials, the Cauchy pressure is a crucial parameter. A positive Cauchy pressure value suggests metallic bonding, while a negative value indicates covalent bonding. The DP materials studied here exhibit positive Cauchy pressure values, as detailed in table 4, and the trend with changing the mixed halogen ratio is portrayed in figure 8b. For polycrystalline materials, this study employs Voigt–Reuss–Hill approximations based on single-crystal elastic constants to evaluate additional mechanical parameters for the double halide perovskites [56,75,76].

To gain a deeper understanding of material rigidity, the bulk modulus (B) is a crucial parameter. Our calculated values, listed in table 4 and illustrated in figure 8a, show that increasing Cl concentration in Cs_2_NaTlBr_6_ perovskite results in a steady increase in bulk modulus from 14.607 GPa for *x* = 0 to 18.487 GPa for *x* = 6. This trend indicates that as Cl content increases, the material becomes stiffer and more resistant to volume changes under pressure, making it flexible and softer. The increase in bulk modulus with higher Cl content is due to Cl’s smaller ionic radius compared with Br, resulting in a more tightly packed, less compressible crystal lattice. Consequently, Cs_2_NaTlBr_6_ is the most flexible and softest in the series, while Cs_2_NaTlCl_6_ is the least flexible and hardest. The tunability of the bulk modulus in Cs_2_NaTlBr_6−*x*_Cl_*x*_ perovskites enhances their versatility for various applications. Lower bulk modulus values (for *x* = 0 and *x* = 1) suggest suitability for flexible electronics and wearable devices, whereas higher values (for *x* = 5 and *x* = 6) provide better structural stability, ideal for rigid photovoltaic panels. In optoelectronic devices like solar cells and LEDs, balancing mechanical flexibility and stability is crucial, and the ability to adjust the bulk modulus by varying the halogen content allows for optimized performance. Additionally, materials with higher bulk modulus are beneficial in acoustic applications requiring efficient sound wave transmission, such as acoustic wave filters and sensors. Young’s modulus (E) reflects a material’s ability to resist longitudinal stress [77]. As shown in table 5, the value of Young’s modulus increases in tandem with the bulk modulus. Specifically, the substitution of Br with Cl in the X site leads to a higher Young’s modulus, indicating enhanced stiffness. This correlation suggests that increasing the Cl content results in a material that is more resistant to deformation under tensile stress. Such materials, with higher Young’s modulus, are ideal for applications requiring rigidity and mechanical strength, such as structural components in photovoltaic panels and optoelectronic devices. Pugh’s ratio (B/G) and Poisson ratio (*v*) are assessed to determine the likely mode of failure (material’s failure mechanism) and listed in table 4 and represented in figure 8b for the Cs_2_NaTlBr_6−*x*_Cl_*x*_ (*x* = 0–6) mixed halide DPs. The ductile–brittle transition of these parameters indicates that a Pugh’s ratio greater than 1.75 suggests ductility, while a Poisson’s ratio exceeding 0.26 also signifies a ductile nature [55] and makes them suitable for the fabrication of flexible devices such as thin films and geometry-optimized optoelectronic devices. Considering the above criterion, from figure 8b, it is evident that as the Cl content increases, the B/G ratio generally decreases, with values ranging from 1.885 for *x* = 0 to 1.865 for *x* = 6. This trend indicates a slight shift towards brittleness as the Cl content increases. Besides, Poisson’s ratio values for the perovskites show a mix of trends, with values ranging from 0.275 to 0.345 for the compositions. Notably, higher Cl content results in a Poisson’s ratio that suggests a transition towards brittleness, particularly evident in the anomalously low value of 0.039 observed for *x* = 5. The perovskites with higher Br content (lower x values) exhibit more ductile behaviour, making them suitable for applications where mechanical flexibility and toughness are essential. Conversely, increasing Cl content results in progressively more brittle materials, which may be advantageous for applications requiring higher stiffness and structural integrity.

**Table 5.** Calculated bulk modulus (B), shear modulus (G), young modulus (E), Poisson’s ratio, bulk/shear ratio (B/G) according to Voigt, Reuss and Hill method, and also calculated Cauchy pressure (GPa), universal elastic anisotropy (A^U^), longitudinal wave velocity (V_l_), transverse wave velocity (V_t_), average wave velocity (V_a_), Debye temperature of Cs_2_NaTlBr_6−x_Cl_*x*_ compounds.

properties	*x* = **0**	*x* = **1**	*x* = **2**	*x* = **3**	*x* = **4**	*x* = **5**	*x* = **6**
B_V_ = B_R_ = B_H_	14.607	15.128	15.722	16.251	17.006	17.449	18.487, 22.77 [50]
G_V_	7.750, 10.98 [29]	8.007	8.204	8.488	9.072	6.048	10.208
G_R_	7.237, 7.82 [29]	7.539	7.842	8.161	8.730	23.190	9.914
G_H_	7.493	7.773	8.023	8.325	8.901	14.619	10.061, 7.86 [50]
E_V_	19.755, 11.51 [29]	20.419	20.965	21.689	23.106	16.265	25.863
E_R_	18.634, 9.98 [29]	19.396	20.173	20.973	22.363	48.209	25.231
E_H_	19.197, 10.57 [29]	19.910	20.570	21.332	22.736	34.283	25.548, 21.15 [50]
Poisson’s ratio, V	0.275	0.275	0.278	0.278	0.274	0.345	0.267
Poisson’s ratio, R	0.287	0.286	0.286	0.285	0.281	0.039	0.273
Poisson’s ratio, H	0.281, 0.26 [50],0.28 [29]	0.281	0.282	0.281	0.277	0.173	0.270, 0.35 [50]
(B/G)_V_	1.885	1.889	1.916	1.914	1.875	2.886	1.811
(B/G)_R_	2.018, 2.11 [29]	2.007	2.005	1.991	1.948	0.752	1.865
(B/G)_H_	1.949	1.946	1.960	1.952	1.911	1.194	1.865
Cauchy pressure (GPa)	1.691	1.987	1.881	1.572	1.971	11.202	1.474
A^U^	0.354, 0.45 [29]	0.310	0.230	0.200	0.196	−3.696	0.148, 0.38 [50]
V_l_	2397.061	2466.354	2538.477	2615.306	2722.284	3125.500	2950.782
V_t_	1323.025	1361.911	1398.888	1442.870	1511.450	1966.168	1657.105
V_a_	1474.279	1517.554	1559.003	1607.878	1683.465	2164.386	1844.013
Debye temp. (Θ_D_)	130.870, 165 [50]	135.862	140.841	146.552	154.902	201.001	172.937, 154 [50]

As listed in table 5 and depicted in figure 8c, the global anisotropy (A^U^) decreases from 0.354 for *x* = 0 to 0.148 for *x* = 6, indicating an anisotropic nature and uniform mechanical response with increasing Cl content. The average wave velocity (V_a_) also increases, rising from 1474.279 m s^−1^ for *x* = 0 to 1844.013 m s^−1^ for *x* = 6, reflecting enhanced stiffness and faster elastic wave propagation. The Debye temperature (Θ_D_) is also calculated and presented in figure 8c and table 5. In crystal thermodynamics, the Debye temperature is crucial as it reflects the temperature at which a crystal’s usual mode of vibration impacts its elasticity and thermodynamic properties. The thermal conductivity coefficient, influenced by phonon vibrations, directly correlates with the Debye temperature and phonon–phonon scattering. Debye temperature also affects the dynamic behaviour of dislocations, with higher values indicating increased interaction between dislocations and conduction electrons, potentially leading to superconducting transitions and significant quantum effects such as quantum tunnelling and oscillations [78–80]. Correspondingly, the Debye temperature (Θ_D_) shows an upward trend, from 130.870 K for *x* = 0 to 172.937 K for *x* = 6, suggesting stronger atomic bonding and improved thermal conductivity in Cl-dominant phases. These trends highlight the potential of these perovskites for applications requiring robust mechanical properties and efficient thermal management, such as in high-performance optoelectronic devices and thermal sensors.

## 3. Conclusions

The comprehensive investigation of the impact of mixed halide phases for Cs_2_NaTlBr_6−*x*_Cl_*x*_ (*x* = 0−6) DPs reveals significant insights into their physical, optoelectronic and mechanical properties, highlighting their potential for various optoelectronic applications. We observed that mixing halogen sites with lower ionic radii (Cl < Br) and changing the ratio results in perovskite compounds with visible-region energy bandgaps (GGA-PBE: 0.80−1.75 eV, HSE06: 1.78−2.98 eV), enhancing their potential for solar cells and optoelectronic devices like UV photodetectors and LED technologies. Calculations using GGA-PBE and HSE06 functionals demonstrate that all phases possess direct bandgaps except *x* = 5. Br-to-Cl substitution reduces crystal symmetry to the Amm2 space group, suggesting ferroelectric behaviour that allows for polar distortions, while real part dielectric analysis (ε₁(0)) also reveals strong optical responses and potential polarization effects. Additionally, mechanical and elastic anisotropy highlight direction-dependent responses, which may hint at ferroelectric distortions in certain compositions. Future investigations are necessary to confirm ferroelectric properties in these perovskites and utilization in polarization-dependent electron-photon dynamics in the NaTl-based perovskites. Our analysis of the optical properties of the studied perovskites suggests that mixed halide-enhanced absorption coefficient and tuned other optical behaviour are beneficial for solar cells, optoelectronics and energy harvesting applications [81–83]. The high carrier mobility observed in Br-dominant compositions supports their integration into high-speed optoelectronic and transistor-based devices, while the increasing bulk modulus and Debye temperature with Cl incorporation make them ideal for wearable and high-temperature device applications. Additionally, the ductile nature of these double halide perovskites makes them suitable for fabricating flexible devices such as thin films and shape-dependent optoelectronic devices. The study reveals that increasing Cl content in the perovskite series enhances both bulk modulus (B) and Young’s modulus (E), indicating greater stiffness due to Cl’s smaller ionic radius than Br. This trend supports the materials’ ductile behaviour with a B/G ratio exceeding 1.75 and Poisson’s ratio (ν) above 0.26, making them promising for flexible device applications.

## Data Availability

Data is available in Dryad [84]. Supplementary material is available online [85].
